# Role of protein arginine methyltransferase 5 in group 3 (MYC-driven) Medulloblastoma

**DOI:** 10.1186/s12885-019-6291-z

**Published:** 2019-11-06

**Authors:** Nagendra K. Chaturvedi, Sidharth Mahapatra, Varun Kesherwani, Matthew J. Kling, Mamta Shukla, Sutapa Ray, Ranjana Kanchan, Naveenkumar Perumal, Timothy R. McGuire, J. Graham Sharp, Shantaram S. Joshi, Don W. Coulter

**Affiliations:** 1 Department of Pediatrics, Division of Hematology and Oncology, University of Nebraska Medical Center, Omaha, NE 68198 USA; 2 Department of Biochemistry and Molecular Biology, University of Nebraska Medical Center, Omaha, NE 68198 USA; 3 Child Health Research Institute Cancer, University of Nebraska Medical Center, Omaha, NE 68198 USA; 4 Department of Genetics, Cell Biology and Anatomy, University of Nebraska Medical Center, Omaha, NE 68198 USA; 5 Department of Pharmacy Practice, University of Nebraska Medical Center, Omaha, NE 69198 USA

**Keywords:** Medulloblastoma, PRMT5, MYC protein, PRMT5 inhibitor

## Abstract

**Background:**

MYC amplification or overexpression is common in Group 3 medulloblastoma and is associated with the worst prognosis. Recently, protein arginine methyl transferase (PRMT) 5 expression has been closely associated with aberrant MYC function in various cancers, including brain tumors such as glioblastoma. However, the role of PRMT5 and its association with MYC in medulloblastoma have not been explored. Here, we report the role of PRMT5 as a novel regulator of MYC and implicate PRMT5 as a potential therapeutic target in MYC-driven medulloblastoma.

**Methods:**

Expression and association between PRMT5 and MYC in primary medulloblastoma tumors were investigated using publicly available databases. Expression levels of PRMT5 protein were also examined using medulloblastoma cell lines and primary tumors by western blotting and immunohistochemistry, respectively. Using MYC-driven medulloblastoma cells, we examined the physical interaction between PRMT5 and MYC by co-immunoprecipitation and co-localization experiments. To determine the functional role of PRMT5 in MYC-driven medulloblastoma, PRMT5 was knocked-down in MYC-amplified cells using siRNA and the consequences of knockdown on cell growth and MYC expression/stability were investigated. In vitro therapeutic potential of PRMT5 in medulloblastoma was also evaluated using a small molecule inhibitor, EPZ015666.

**Results:**

We observed overexpression of PRMT5 in MYC-driven primary medulloblastoma tumors and cell lines compared to non-MYC medulloblastoma tumors and adjacent normal tissues. We also found that high expression of PRMT5 is inversely correlated with patient survival. Knockdown of PRMT5 using siRNA in MYC-driven medulloblastoma cells significantly decreased cell growth and MYC expression. Mechanistically, we found that PRMT5 physically associated with MYC by direct protein-protein interaction. In addition, a cycloheximide chase experiment showed that PRMT5 post-translationally regulated MYC stability. In the context of therapeutics, we observed dose-dependent efficacy of PRMT5 inhibitor EPZ015666 in suppressing cell growth and inducing apoptosis in MYC-driven medulloblastoma cells. Further, the expression levels of PRMT5 and MYC protein were downregulated upon EPZ015666 treatment. We also observed a superior efficacy of this inhibitor against MYC-amplified medulloblastoma cells compared to non-MYC-amplified medulloblastoma cells, indicating specificity.

**Conclusion:**

Our results reveal the regulation of MYC oncoprotein by PRMT5 and suggest that targeting PRMT5 could be a potential therapeutic strategy for MYC-driven medulloblastoma.

## Background

Medulloblastoma is the most common malignant pediatric brain tumor, accounting for nearly 20% of all childhood brain cancers [1]. Current therapies of medulloblastoma have improved patient survival to about 70% and include surgical resection, radiation therapy, and chemotherapy [2]. Medulloblastoma has biological/genetic heterogeneity with 4 major molecularly distinct subgroups including wingless (WNT), Sonic Hedgehog (SHH), Group 3 and Group 4 [3–5]. Group 3 medulloblastoma often exhibits MYC amplification or overexpression and has the worst prognosis of the 4 medulloblastoma subgroups with < 50% survival. MYC-driven medulloblastomas have high metastatic potential and are often resistant to even multimodal treatments [6–8]. Thus, understanding the mechanisms of MYC-driven tumor progression/recurrence and integration of molecular-targeted therapies are critical to identifying novel and effective therapeutics for these high-risk patients.

Epigenetic deregulation has emerged as a key driver in medulloblastoma tumorigenesis, particularly alterations in histone modifying enzymes such as histone methyl transferases [9, 10]. Furthermore, Group 3 and Group 4 medulloblastomas present with high levels of histone H3-lysine 27 tri-methylation (H3K27me3) due to altered activity of the H3K27 methyltransferase and H3K27 demethylases [11, 12]. Post-translational methylation of histone may occur at lysine (K) or arginine (R) residues. Past studies have focused more on histone lysine methylation than histone arginine methylation. However, growing evidence supports the importance of arginine methylation by protein arginine methyltransferases (PRMTs) in cancer progression. Particularly, the overexpression of PRMT5 has been correlated with poor prognosis in a variety of cancers [13].

PRMT5 represents a member of PRMT family proteins that methylate histone and non-histone proteins to regulate gene expression and cellular development [14]. PRMT5 symmetrically dimethylates the arginine residues of histone proteins H4 (S2Me-H4R3), H3 (S2Me-H3R8) and H2A, and thereby regulates chromatin structure to support transcriptional repression [15]. PRMT5 over-expression in cancers is thought to epigenetically silence tumor suppressor and cell cycle genes [16]. In addition, PRMT5 is known to post-translationally methylate certain oncogenic transcription factors (non-histone proteins) such as p53, NF-κB (p65) and MYCN [17–20].

Recently, PRMT5 was found to associate with aberrant MYC function in various cancers including brain tumors such as glioblastoma and neuroblastoma [20–23]. However, the role of PRMT5 and its association with MYC in medulloblastoma have not been explored. Based on these observations, we hypothesized that PRMT5 is a novel regulator of MYC expression whose inhibition may serve as a novel therapeutic strategy in MYC-driven medulloblastoma.

## Methods

### Cell lines and culture

The human medulloblastoma cell lines Daoy (HTB-186), D-283 (HTB-185) and D-341 (HTB-187) were purchased from American Type Culture Collection (ATCC, Manassas, VA, USA). HD-MB03 (ACC-740) human medulloblastoma cell line was purchased from Deutsche Sammlung von Mikroorganismen und Zellkulturen (DSMZ, Braunschweig, Germany). ONS-76 (IFO50355) human medulloblastoma cell line was obtained from Sekisui-XenoTech (Kansas, USA). These Cell lines were authenticated by their respective companies using short tandem repeat profiling. All cell lines were tested for mycoplasma contamination using MycoSensor PCR Assay Kit (Santa Clara, CA, USA). In this study, Daoy and ONS-76 were used as SHH medulloblastoma subgroup cell lines without MYC-amplification, whereas, D-341, HD-MB03 and D-283 were used as Group 3 medulloblastoma cell lines with MYC-amplified status. All these cell lines were cultured and maintained using Eagle’s minimal essential medium (EMEM) or RPMI-1640 media supplemented with 10% heat-inactivated FBS and 1% penicillin/streptomycin (Invitrogen, Carlsbad, CA, USA) in a humidified incubator at 5% CO2 and 95% air atmosphere at 37 °C. The experiments were performed using no more than 10 passages for each cell line. The cell lysate of human normal brain cerebellum was purchased from BioChain Institute Inc. (Newark, CA, USA).

### Patient data acquisition for PRMT5 expression and survival

The R2 Genomics Analysis and Visualization Platform (www.r2.amc.nl) was used to investigate *PRMT5* mRNA expression and its correlation with patient survival across medulloblastoma subgroups using publicly available datasets. The expression of *PRMT5* mRNA in medulloblastoma was analyzed using a total 491 medulloblastoma tumors (5 independent cohorts) and 9 normal cerebellum samples. The survival analyses with respect to PRMT5 expression in medulloblastoma patients were performed using a separate cohort of 612 medulloblastoma samples from Cavalli (763 samples) dataset.

### siRNAs and inhibitor

Both control (Scrambled, sc-37,007) and PRMT5 siRNAs ((sc-41,073) were purchased from Santacruz Biotechnology (Dallas, TX, USA). Each siRNA was dissolved in RNase-free water at 10 μM stock concentration and stored at -20 °C. The PRMT5 inhibitor EPZ015666 was purchased from Selleckchem Company (Houston, TX, USA). This inhibitor was dissolved in DMSO at 10 mM stock concentration and stored at -20 °C.

### siRNA knock-down and transfection

Control (scrambled) and PRMT5 siRNA (a pool of 3 target-specific 19–25 nt siRNAs with 50 nM) were transiently transfected into medulloblastoma cells using Lipofectamine 2000 (Invitrogen, Carlsbad, CA, USA) according to the manufacturer’s instructions. Following 72 h of transfections, cells were subjected to downstream analyses using western blotting and MTT assay.

### Cell growth assay

To examine the effects of PRMT5 inhibition on medulloblastoma cell growth, twenty thousand cells of each medulloblastoma cell line were plated in 96-well plates 24 h before the experiment. Then, these cells were transfected with PRMT5 siRNAs or treated with PRMT5 inhibitor for 72 h according to the experimental plan and the growth of these cells was determined using an MTT assay as described previously [24].

### Apoptosis and cell cycle analyses

The effect of PRMT5 inhibitor to induce apoptosis in medulloblastoma cells at 72 h, was determined using an Annexin-V:FITC flow cytometry assay kit (BD Biosciences, San Jose, CA, USA) following the manufacturer’s instructions. For cell cycle analysis, the control and PRMT5 inhibitor-treated medulloblastoma cells for 24 and 48 h, were fixed with 75% ethanol and stained with propidium iodide using a propidium iodide flow cytometry kit (Abcam, Cambridge, UK).

### Cycloheximide chase and co-immunoprecipitation experiments

To determine protein stability, medulloblastoma cells were treated with 50 μg/ml cycloheximide (Sigma Aldrich, St. Louis, MO, USA) following siRNA transfection for 72 h. Following transfection, cell lysates from the indicated time points of cycloheximide treatments were subjected to western blotting.

For co-immunoprecipitation, 500 μg protein lysate was precleared with 50 μl of protein A-Sepharose beads (Cell Signaling Technology, Danvers, MA, USA) for 1 h at 4 °C. Immunoprecipitation was performed in the presence of 8 μg of the indicated primary antibodies at 4 °C overnight. Immune complexes were captured by adding 50 μl of protein A-Sepharose beads and rotated at 4 °C for 2 h. After the supernatant was discarded, protein A-Sepharose beads were washed with PBS and lysed in 1x Laemmli buffer and then subjected to western blotting.

### Western blotting

The expression levels of indicated proteins in medulloblastoma cells were determined using western blot analyses as described previously [24]. The primary human antibodies for cMYC (sc-40), PRMT5 (sc-376,937), histone H3 (sc-8654) and β-Actin (sc-130,301) were purchased from Santacruz Biotechnology (Dallas, TX, USA). H4R3me2s (61188) and H3R8me2s (ab130740) antibodies were from Active Motif (Carlsbad, CA, USA) and Abcam (Cambridge, UK), respectively. Immunoreactivity was detected using appropriate peroxidase-conjugated secondary antibodies (Jackson Lab, ME) and visualized using an ECL detection system (Pierce, IL).

### Immunofluorescence

Methanol-fixed HD-MB03 cells on glass cover slips, and an antigen-retrieved medulloblastoma tumor section were washed with PBS and blocked in 1% BSA in PBS for 30 min. The tumor cells were then co-incubated with PRMT5 (rabbit, 1:100) and MYC (mouse, 1:100) antibodies overnight at 4 °C. Following three washes with PBS, the cells were further co-incubated with fluorochrome-conjugated anti-rabbit (Alexa-488) and anti-mouse (Alexa-647) secondary antibodies (Invitrogen, Carlsbad, CA) for 1 h at room temperature. The cells were then washed three times with PBS and the cover slips were mounted on glass slides and visualized under confocal microscope. DAPI was co-incubated with the secondary antibodies to facilitate the visualization of the nuclei. Confocal images were taken using a Zeiss LSM 5 Pascal confocal microscope (Carl Zeiss, Oberkochen, Germany) using a 40x objective in the UNMC Confocal Microscopy facility.

### Immunohistochemical analyses in patient samples

Frozen samples of normal cerebella and medulloblastoma tumor specimens were collected from the Children’s Hospital and Medical Center, Omaha and the University of Nebraska Medical Center after Institutional Review Board (IRB) approval. Normal cerebellum specimens were obtained from patients at autopsy. All normal and tumor samples were from the pediatric age group.

Normal cerebellum and medulloblastoma tumor sections were deparaffinized with xylene and rehydrated with water. Antigen retrieval was performed using citrate buffer at 95 °C for 20 min. Sections were treated with 3% hydrogen-peroxide for 30 min to block peroxidase activity. Sections were blocked using 5% goat serum with 0.3% Triton-X-100 in PBS and incubated with PRMT5 (1:100) and MYC (1:100) rabbit-antibodies (Abcam, Cambridge, UK) overnight at 4 °C. Next day, primary antibodies were washed with PBS three times and incubated with appropriate HRP-conjugated secondary antibodies for 1 h at room temperature. Following three washes with PBS, detection was performed using a DAB Peroxidase Substrate Kit (Vector Labs, Burlingame, CA, USA) followed by counterstaining with hematoxylin. Sections were mounted in Paramount solution and visualized under an EVOS FL Auto Imaging System (Life Technologies, Carlsbad, CA, USA). Staining intensity was scored from 0 to 3, where signal detected at 10X was 3+, at 20X was 2+, at 40X was 1+, and no detection was 0. The percentage positive cells was scored from 1 to 4 scale, where < 25% scored 1, 25–50% scored 2, 50–75% scored 3; and > 75 scored 4. Composite score (0–12) was derived from the staining intensity and % positive cells.

### Statistical analysis

All experiments were repeated at least two times and the mean and standard error values calculated. Differences (*p*-value) were calculated using independent Student t-tests or analysis of variance (ANOVA) and *p*-values < 0.05 were considered significant. The IC_50_ values of inhibitor EPZ015666 for each medulloblastoma cell line were determined using GraphPad Prism V6 software (provided in Table 1).

**Table 1:** IC_50_ of EPZ015666 in medulloblastoma cell lines (MTT assay 72 h)

MB Cell Line	IC_50_ (μM)
Daoy	> 10
ONS-76	> 10
D-283	4.87
D-341	1.72
HD-MB03	2.44

## Results

### PRMT5 expression correlates with MYC in primary medulloblastoma and cell lines

**Fig. 1:**
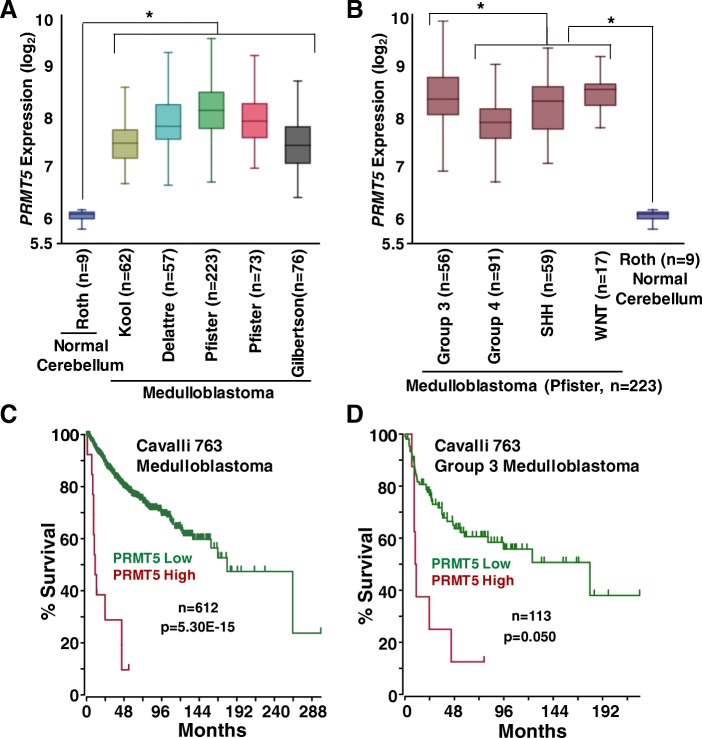
*PRMT5* expression and correlation in primary medulloblastomas. **a** Boxplots showing *PRMT5* expression in five non-overlapping cohorts (total *n* = 491) of medulloblastoma tumors compared to normal cerebellum (*n* = 9) controls. *Anova *p* < 0.05 vs. medulloblastoma. **b** *PRMT5 expression* in four (Group3, Group 4, SHH and WNT) medulloblastoma subgroups using Pfister (*n* = 223) cohort dataset. *Anova *p* < 0.05 vs. Group 3. Kaplan-Meier plots showing overall survival of patients (Cavalli 763 cohort) with medulloblastoma all subgroups (**c**) and Group 3 medulloblastoma (**d**) with respect to *PRMT5* expression

The aberrant expression of PRMT5 has been associated with a variety of cancers including glioblastoma and neuroblastoma. In addition, PRMT5 expression has correlated with MYC or MYCN protein in these cancers [20–23]. However, its expression and function in medulloblastoma have not been reported. These studies prompted us to examine the correlation between MYC levels and PRMT5 expression in medulloblastoma. We first examined the clinical relevancy of PRMT5 in medulloblastoma by analyzing its mRNA expression in 491 medulloblastoma (from independent 5 cohorts) and 9 normal cerebellum samples using the R2 platform (www.r2.amc.nl). Our analyses using these data showed a significant overexpression of *PRMT5* in medulloblastoma compared to normal cerebellum tissues (Fig. 1a). We further analyzed the *PRMT5* expression across medulloblastoma subgroups using a cohort that has maximum number (223) of samples with all 4 molecular subgroups. We observed significantly higher expression of *PRMT5* in Group 3 (MYC-driven) medulloblastoma compared to other 3 subgroups (Fig. 1b). We next compared PRMT5 expression against patient survival. To this end, we performed survival analyses with respect to *PRMT5* expression, using 612 medulloblastoma samples from the Cavalli (763 samples) dataset. Our survival analyses showed that high levels of *PRMT5* expression correlated with poor survival of medulloblastoma patients, a pattern recapitulated in Group 3 medulloblastoma patients (Fig. 1c and d). These data suggest that PRMT5 expression is not simply deregulated in medulloblastoma, but is also a poor prognostic marker, particularly in Group 3 (MYC-driven) tumors.

**Fig. 2:**
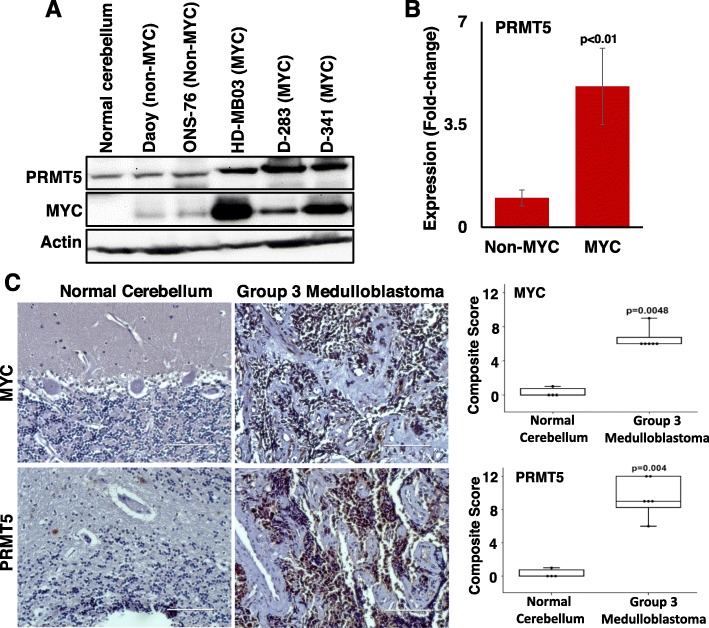
Expression and correlation of PRMT5 with MYC protein in medulloblastoma cell lines and primary tumors. **a** Western blotting of PRMT5 in medulloblastoma cell lines with and without MYC amplification compared to normal cerebellum. Actin was used as a loading control. **b** Comparison of PRMT5 expression levels normalized to Actin in non-MYC vs MYC amplified medulloblastoma cell lines. Significance, *p* < 0.01. **c** Representative immunohistochemical images showing PRMT5 and MYC expression and their localization pattern in normal pediatric cerebellum and Group 3 medulloblastoma tumor tissues at 20x magnification. Scale bar, 200 μm. The box plots on right side showing quantification of composite score-based intensity of MYC and PRMT5 staining in Group 3 medulloblastoma tumor specimen (*n* = 6) compared to normal pediatric cerebellum (*n* = 4)

We next analyzed the correlation between PRMT5 and MYC mRNA expression across medulloblastoma subgroups using Pfister (*n* = 223) cohort at the R2 genomic analysis platform. Results from this analysis showed that high expression of PRMT5 was strongly correlated (R-value = 0.531; *p*-value = 2.51e-05) with high MYC in Group 3 medulloblastoma. Although results showed some degree of correlation (R-value = 0.069–0.284; p-value = 0.111–0.606) of these genes in the other three medulloblastoma subgroups, none of these medulloblastoma subgroups showed a significant correlation between PRMT5 and MYC expression (Additional file 1: Figure S1). We further examined the correlation between PRMT5 and MYC expression at the protein levels by western blotting in non-MYC (Daoy, ONS-76) and three MYC-driven (D-283, D-341, HD-MB-03) medulloblastoma cell lines compared to normal cerebellum. We observed that PRMT5 protein levels were significantly (*p* < 0.01) higher in MYC-driven medulloblastoma cell lines compared to non–MYC medulloblastoma cell lines and normal human cerebellum cells (Fig. 2a and b). Stronger PRMT5 band intensity seemed associated with higher MYC expression in medulloblastoma cell lines. To further authenticate this correlation at the protein level, we examined the expression of PRMT5 and MYC by immunohistochemistry in Group 3 medulloblastoma tumor samples (*n* = 6) compared to normal pediatric cerebellum (*n* = 4). We found that the protein levels of PRMT5 and MYC were significantly (*p* = 0.004) higher with more than 75% staining in Group 3 medulloblastoma samples than normal pediatric cerebellum tissues (Fig. 2c). We observed very poor immunostaining of these proteins in normal cerebellum with less than ‘1’ intensity score. We not only observed intensely high expression of PRMT5 and MYC protein in Group 3 medulloblastoma but also, a positive correlation with their predominantly nuclear co-expression. These results consistently suggest a positive correlation and co-operative role of the PRMT5-MYC oncogenic axis in poor prognosis medulloblastoma.

### PRMT5 knockdown leads to decreased MYC expression and cell survival in MYC-driven medulloblastoma cells

**Fig. 3:**
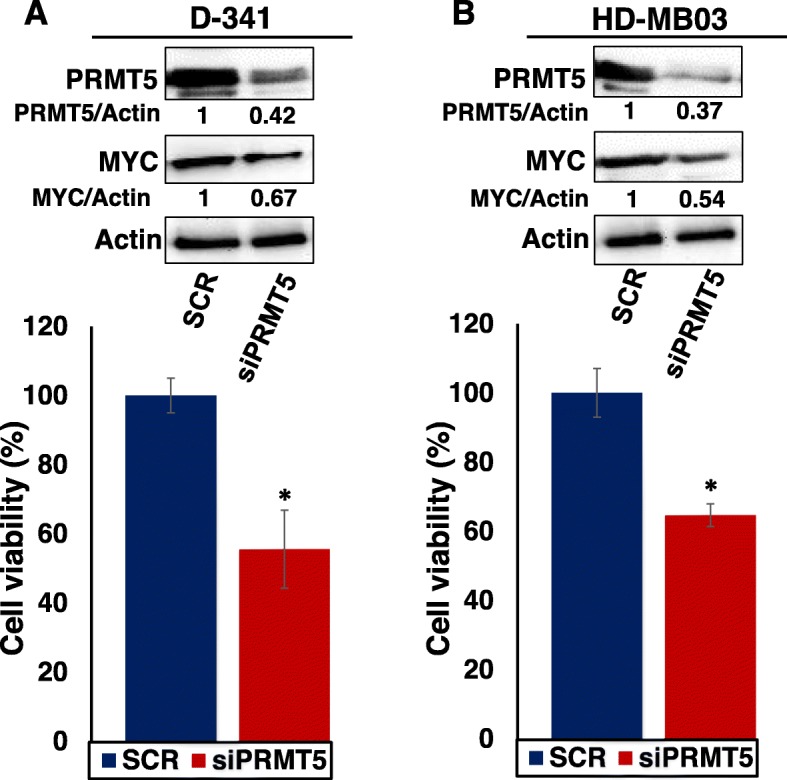
PRMT5 knockdown in MYC-driven medulloblastoma cells**.** MYC-amplified medulloblastoma cell lines D-341 (**a**) and HD-MB03 (**b**) were transiently transfected with PRMT5-siRNA and control scrambled siRNA (SCR) for 72 h. Following transfections, cells were subjected to cell growth analyses using MTT assay and western blotting to determine the expression levels of PRMT5 and MYC proteins. The values given below each western blot are showing the densitometric quantification of each protein expression relative to the control SCR after Actin normalization. *, *p* < 0.05

The strong correlation between PRMT5 and MYC expression prompted us to explore possible influence of PRMT5 on MYC function. To investigate the role of PRMT5 on MYC function, we determined the effect of short-interfering RNA (siRNA)-mediated knockdown of PRMT5 on MYC expression and cell survival in MYC-amplified medulloblastoma cell lines. We used two cell lines D-341 and HD-MB03 in this study as both lines have previously been reported as well-established Group 3 medulloblastoma cell lines with high MYC expression. Consistently, our western blot results further confirmed strongly higher expression of MYC protein in these two cell lines compared to other medulloblastoma lines (Fig. 2a). Using these two lines, we first verified the knockdown of PRMT5 protein expression after siRNA transfections and then assessed the consequences of knockdown. We found that knockdown of PRMT5 efficiently suppressed the expression of MYC protein in both cell lines by approximately 35–45%, compared to control scrambled siRNA (Fig. 3), suggesting an on-target effect of PRMT5. Concurrently, PRMT5 knockdown significantly reduced cell growth (Fig. 3) in both cell lines by approximately 40–45%, compared to control scrambled siRNA. We also observed that D-341 cell line showed relatively lesser knockdown of PRMT5 and MYC protein by 5 and 13%, respectively, compared to HD-MB03 cell line. However, the impact of knockdown on inhibition of D-341 cell growth was relatively (~ 5%) higher compared to HD-MB03 cell growth, indicating differential knockdown activity between two MYC-amplified cell lines. One possible explanation for this differential response in cell lines could be that they have different growth pattern in culture in vitro*,* as we observed that D-341 cells grow more slower with mixed spheroids and monolayer cells compared to mostly monolayer HD-MB03 cells.

### Physical and functional interaction of PRMT5 and MYC in MYC-driven medulloblastoma cells

**Fig. 4:**
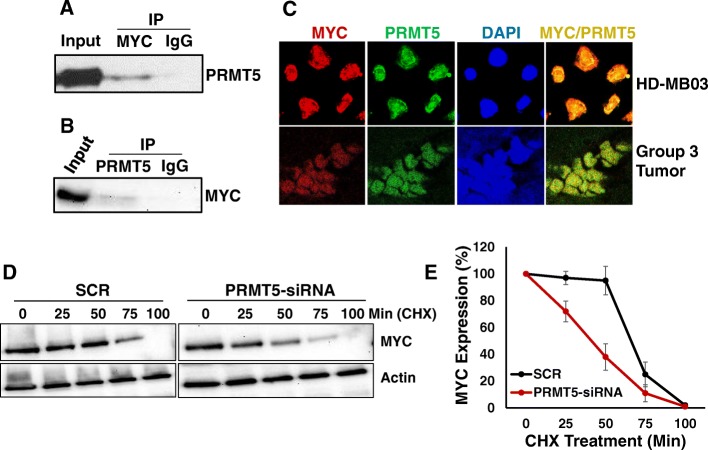
Physical and functional interaction between PRMT5 and MYC. **a** HD-MB03 cell lysate was subjected to co-immunoprecipitation (IP) analysis using MYC antibody and control IgG, followed by immunoblotting with PRMT5 antibody. **b** HD-MB03 cell lysate was subjected to reciprocal co-immunoprecipitation using PRMT5 antibody and control IgG, followed by immunoblotting with MYC antibody. **c** Confocal images for the co-localization of MYC and PRMT5 in HD-MB03 cells and Group 3 medulloblastoma tumor tissue at 40x magnification. **d** Western blot analysis of MYC expression after 50 μg/ml CHX treatment following transient transfection of scrambled siRNA (SCR) and PRMT5-siRNA in HD-MB03 cells. **e** Densitometric quantification of MYC protein expression shown in “d”

Previous studies [20, 23] in neuroblastoma and glioblastoma have demonstrated that PRMT5 can physically interact with MYC and regulate its stability at the post-translational level. To confirm whether endogenous PRMT5 physically interacts with MYC protein in medulloblastoma, we performed a co-immunoprecipitation experiment in MYC-driven HD-MB-03 medulloblastoma cells using MYC and PRMT5 antibodies. Our results presented in Fig. 4a, showed the presence of PRMT5 in MYC-immunoprecipitated complexes from cell extracts. We further confirmed this interaction by detecting MYC in the reverse PRMT5-immunoprecipitated complexes (Fig. 4b). To further support of association between PRMT5 and MYC, we examined co-localization of these two proteins in HD-MB03 cells and a tumor specimen of a Group 3 medulloblastoma patient, using immunofluorescence-coupled with confocal microscopy. As shown in Fig. 4c, the merged immunofluorescent-staining of MYC and PRMT5 demonstrated a significant co-localization pattern in both HD-MB03 and primary tumor cells. The results further showed that both MYC and PRMT5 were predominantly co-localized in the nucleus, and this localization pattern was consistent with their immunohistochemical co-expression in Group 3 medulloblastoma primary tumors shown in Fig. 2c. Together, the results of co-immunoprecipitation and co-localization of MYC and PRMT5 suggest that endogenous PRMT5 forms a complex with MYC in medulloblastoma cells harboring MYC amplification. The observation of physical interaction between PRMT5 and MYC indicates a potential functional role of this novel protein complex in medulloblastoma.

To determine the consequence of this physical interaction on post-translational MYC stability and influence on MYC expression, we performed cycloheximide (CHX) chase experiments in PRMT5 knocked-down HD-MB03 cells and measured the half-life of MYC protein. As shown in Fig. 4d and e, MYC has a half-life of approximately 60 min after CHX treatment in cells transfected with control scrambled siRNA, whereas PRMT5 knockdown dramatically decreased its half-life to approximately 35 min. There was approximately 25 min earlier degradation of MYC protein in PRMT5 knocked-down cells compared to control siRNA treated cells. Together, these results suggest that PRMT5 physically interacts with and stabilizes MYC in medulloblastoma cells at the post-translation level.

### Anti-medulloblastoma efficacy of a small molecule inhibitor of PRMT5

**Fig. 5:**
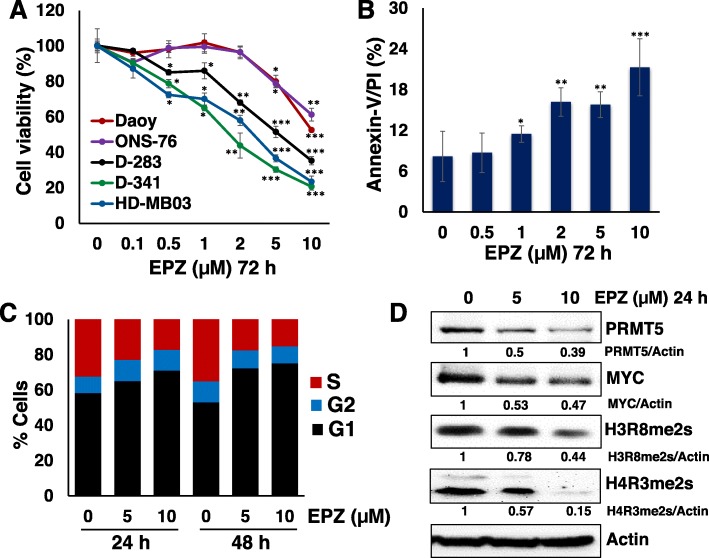
Therapeutic efficacy of PRMT5 inhibitor EPZ015666 in medulloblastoma cell lines. **a** MTT assay showing the dose-dependent effects of EPZ015666 (0.1–10 μM) on non-MYC (Daoy, ONS-76) and MYC-amplified (D-283, HD-MB03, D-341) medulloblastoma cell growth. The values represent the means ± SD from four wells of 96-well plates. The percentage of cell viability is relative to control vehicle-treated cells. *, *p* < 0.05; **, *p* < 0.01, ***, *p* < 0.001 (**b**) Annexin-V assay showing effect of EPZ015666 on apoptosis in HD-MB03 cells. *, *p* < 0.05; **, *p* < 0.01, ***, *p* < 0.001 (relative to ‘0’ (control vehicle). **c** Cell cycle profile in EPZ015666-treated HD-MB03 cells. **d** Western blot analysis for the expression of the indicated key proteins in EPZ015666-treated HD-MB03 cells. The values given below each western blot are showing the densitometric quantification of each protein expression relative to the control after β-Actin normalization

Given the potential for anti-neoplastic effects with PRMT5 knockdown, via reduction in cell viability and MYC expression, we investigated the therapeutic potential of PRMT5 inhibition using a recently developed potent PRMT5 inhibitor (EPZ015666) [25]. We first determined the growth inhibitory efficacy of EPZ015666 against three MYC-amplified (D-283, D-341, HD-MB03) and two non-MYC amplified (Daoy, ONS-76) medulloblastoma cell lines. Cells were treated with inhibitor (0.1–10 μM) in a dose-dependent manner for 72 h and growth of cells was assessed using an MTT assay. Our MTT results clearly demonstrated that EPZ015666 significantly induced the dose-dependent growth inhibition of all MYC-driven medulloblastoma cell lines at low micromolar potency with IC50 of ~ 1.5–2.5 μM (Fig. 5a, Table 1). However, there was minimal effect of EPZ015666 on growth inhibition of non-MYC amplified medulloblastoma cells even at higher doses, suggesting anti-neoplastic specificity of EPZ015666 to MYC-dependent tumors.

We next determined the ability of EPZ015666 to induce apoptosis in a representative MYC-amplified medulloblastoma cell line HD-MB03. The results of the apoptosis analyses (Fig. 5b) using Annexin-V assay demonstrated a dose-dependent induction of apoptosis by EPZ015666 and showed consistency with MTT growth results. Since PRMT5 is known to act during the G1 cell cycle phase, we sought to investigate whether inhibiting PRMT5 by EPZ015666 reduced medulloblastoma cell growth by disrupting the cell cycle. The cell cycle results (Fig. 5c) using PI staining of DNA, demonstrated that treatment of EPZ015666 at 24 h and 48 h arrested medulloblastoma cells in the G1 cell cycle phase in a dose-dependent manner. Since we observed a doubling time of the HD-MB03 cell line of between 28 to 36 h, we examined the impact of EPZ015666 on HD-MB03 cell cycle at 24 and 48 h, close to the doubling time points. Lastly, using western blot analyses, we confirmed that treatment of HD-MB03 cells with optimum doses of EPZ015666 efficiently downregulated the expression levels of PRMT5 and its key target symmetric dimethylated histone H3 (H3R8me2s) and H4 (H4R3me2s), including suppressed MYC expression (Fig. 5d). Taken together, these results suggest that inhibiting the specific interaction of PRMT5 with MYC arrests medulloblastoma cell growth and favors apoptosis in MYC-dependent tumors.

## Discussion

Despite significant improvements in outcomes and overall survival of medulloblastoma patients with current therapies, patients with high-risk disease, particularly MYC-driven medulloblastomas still face a paucity of effective therapies [2]. The minimal improvement in survival of these high-risk medulloblastoma patients identifies a need for novel targeted therapeutic approaches against MYC-driven (high-risk) medulloblastoma. In addition to genetic abnormalities, deregulated epigenetic modifiers are frequently observed in these aggressive medulloblastoma tumors [9, 10]. The importance of epigenetic control in aggressive medulloblastoma underscores the need to identify and understand epigenetic regulatory mechanisms and their targets.

The evolutionarily-conserved PRMT family of enzymes is involved in a wide range of developmental and cellular processes. PRMT5 is the major type II arginine methyltransferase that silences gene transcription by symmetric dimethylation of arginine residues on histone proteins [15, 16]. PRMT5 is involved in the epigenetic regulation of chromatin complexes by interacting with a number of proteins including transcription factors [26]. Growing evidence suggests that PRMT5 expression and activity are dysregulated in various solid and hematological malignancies [16]. Recent studies found PRMT5 as a key epigenetic regulator in glioblastoma tumorigenesis. Interestingly, increased expression of PRMT5 positively correlates with high-grade glioma malignancy and is inversely associated with patient survival. In addition, high levels of MYC and PRMT5 correlate with glioma malignancy [21–23]. Further, PRMT5 is associated with MYCN (another member of the MYC family of transcription factors) in neuroblastoma cells and promotes its stability [20]. However, the role of PRMT5 and its association with MYC in medulloblastoma are unexplored. Here, we showed that PRMT5 is a novel regulator of MYC protein in medulloblastoma.

To address the role of PRMT5 in medulloblastoma, we first accessed expression of PRMT5 across medulloblastoma subgroups including Group 3 medulloblastoma patients and high-MYC expressing medulloblastoma cell lines. Our expression findings confirm that high levels of PRMT5 not only mirror MYC expression in the most aggressive medulloblastomas but also inversely correlate with poor outcomes in patients. This finding purports the clinical utility of PRMT5 as a prognostic marker for patients with more aggressive disease. Although numerous epigenetic abnormalities have been reported in medulloblastoma tumors, including expression of histone and DNA methyltransferases [27], the prognostic applicability of these markers remains unclear. Identification of prognostic markers like PRMT5 will contribute to developing novel therapeutic strategies for this disease.

Our data on PRMT5 knockdown in MYC-amplified medulloblastoma cells showed that PRMT5 can regulate the stability of MYC protein by physically interacting with it, suggesting MYC regulation by PRMT5 at the post-translation level. Further, we showed that knockdown of PRMT5 suppressed medulloblastoma cell growth by inhibiting MYC expression, suggesting a functional role of PRMT5-MYC interaction in medulloblastoma tumorigenesis. These are in agreement with a previous study by Park et al. [20], where they showed similar interactions in neuroblastoma. Since our results showed that PRMT5 and MYC co-expressed and co-localized predominantly in the nucleus, it is possible that PRMT5 may also regulate MYC expression at the transcriptional level. Further studies with the analyses of MYC association to the chromatin and promoter activity are required to explore the possibility of transcriptional regulation of MYC by PRMT5. In addition, it is highly likely that there are other mechanism(s) that could be involved in the PRMT5-mediated regulation of MYC. Such analyses would certainly be a topic for future studies.

PRMT5 is a key and emerging stemness factor for normal and cancer stem cells. Its role in stemness has been demonstrated in embryonic and neural stem cells [28–30]. Given that neural stem or cancer stem cells have profound impact on driving medulloblastoma tumorigenesis and recurrence, there might be role of PRMT5 in regulating self-renewal capacity of medulloblastoma tumor initiating cells. Recently, PRMT5 has also been shown to methylate a key stemness factor KLF4 in breast cancer [31]. Methylation of KLF4 by PRMT5 leads to stabilization of KLF4 protein, resulting in promotion of tumorigenesis. In a subsequent study, the authors developed a novel and potent PRMT5 inhibitor, WX2–43, that disrupts PRMT5-KLF4 interaction and suppresses breast cancer progression [32]. Further investigation of targeting unexplored PRMT5-KLF4 interactions in medulloblastoma might be another new strategy to develop therapy for MYC-driven medulloblastoma.

Given the role of PRMT5 in MYC-driven medulloblastoma cells, we further tested the therapeutic potential of targeting PRMT5 using a selective small molecule inhibitor, EPZ015666, against medulloblastoma cell lines. Our results demonstrated that EPZ015666 significantly inhibits proliferation and survival of MYC-driven medulloblastoma cells associated with G1-S cell cycle arrest. Our results also indicated that MYC-amplified cells show greater sensitivity to EPZ015666 compared to non-MYC amplified medulloblastoma cells, further supporting the role of PRMT5 acting in MYC-dependent manner. Molecularly, EPZ015666 significantly downregulated the expression of PRMT5 and MYC protein in MYC-driven cells. These data support our hypothesis of the potential for PRMT5 to serve as a therapeutic target in MYC-driven medulloblastoma and this warrants further, systematic evaluation in appropriate preclinical mouse models.

## Conclusion

In summary, we have demonstrated for the first time that PRMT5 is a critical regulator of MYC expression in MYC-amplified medulloblastomas. PRMT5 and MYC expression are positively correlated in medulloblastoma cells. Mechanistic studies revealed that PRMT5 could elevate MYC expression and stability, enhancing medulloblastoma tumorigenicity. Our results using a PRMT5 inhibitor EPZ015666 highlight the PRMT5-MYC oncogenic axis a viable therapeutic approach for MYC-driven medulloblastoma. With evaluation of this approach in preclinical mouse models, we may take the first steps towards translating this discovery to the clinic.

## Supplementary information

**Additional file 1: Figure S1.** The expression correlation of *PRMT5*gene with *MYC*gene in medulloblastoma. These data were analyzed using a Pifster(*n* = 223) cohort at R2-Genomics platform.

## Data Availability

All data generated or analyzed during this study are included in this article.

## Abbreviations

DNA: Deoxyribonucleic acid
FBS: Fetal bovine serum
FITC: Fluorescein isothiocyanate
h: Hour
IC_50_: Inhibitory concentration of inhibitor with 50% inhibition;
min: Minute
ml: Milliliter
mRNA: Messenger ribonucleic acid
MTT: 3-(4,5-dimethylthiazol-2-yl)-2,5-diphenyltetrazolium bromide
MYC: v-myc avian myelocytomatosis viral oncogene homolog
nm: Nanometer
nt: Nucleotide
PBS: Phosphate buffered saline
SDS: Sodium dodecyl sulfate
siRNA: Small interfering ribonucleic acid
μg: Microgram
μl: Microliter
μm: Micrometer
μM: Micromolar
