# Supplementary material for: Role of protein arginine methyltransferase 5 in group 3 (MYC-driven) Medulloblastoma
Source: BMC Cancer. 2019 Nov 6;19:1056. doi: 10.1186/s12885-019-6291-z (PMC6836472; doi:10.1186/s12885-019-6291-z)
Supplement: Supplementary file 1 — Additional file 1: Figure S1. The expression correlation of PRMT5gene with MYCgene in medulloblastoma. These data were analyzed using a Pifster(n = 223) cohort at R2-Genomics platform. [file 12885_2019_6291_MOESM1_ESM.pdf]

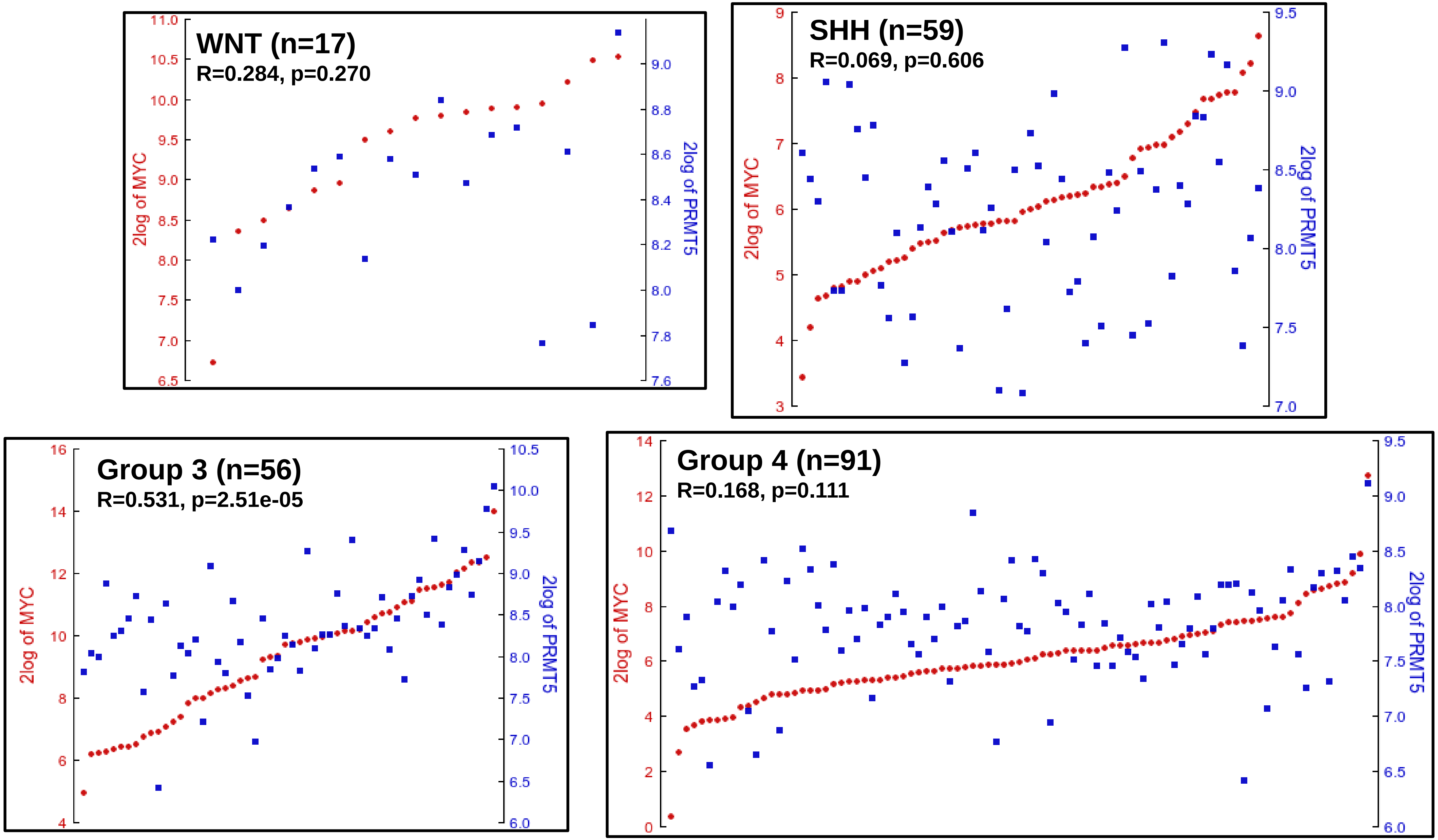

Fig. S1. The expression correlation of *PRMT5* gene with *MYC* gene in medulloblastoma. These data were analyzed using a Pifster (n=223) cohort at R2-Genomics platform.
